# Rare Anal Canal Cancer with Secondary Extramammary Paget's Disease (Pagetoid Spread) Complicated by Squamous Cell Carcinoma of the Skin

**DOI:** 10.1155/2021/9944886

**Published:** 2021-07-30

**Authors:** Tatsuki Kusuhara, Takashi Ito, Hiroki Matsuoka, Teruhiro Chohno, Masataka Zozumi, Takamasa Ohnishi

**Affiliations:** ^1^Department of Surgery, Nishiwaki Municipal Hospital, 652-1 Shimotoda, Nishiwaki, Hyogo 677-0043, Japan; ^2^Department of Diagnostic Pathology, Nishiwaki Municipal Hospital, 652-1 Shimotoda, Nishiwaki, Hyogo 677-0043, Japan

## Abstract

A 91-year-old man had a node and erythema in the anal area resistant to treatment. A biopsy of the node in the anus showed atypical cells developing as Paget's disease, and staining revealed that the cells were CK7-positive, CK20-positive, and GCDFP15-negative. Therefore, tumor invasion with pagetoid spread (PS) from the anus to the skin was suspected, and the patient was referred to our department for a close examination and surgical treatment. Lower gastrointestinal endoscopy showed edematous, hemorrhagic mucosa in the anal canal, and he was diagnosed with adenocarcinoma via a biopsy. Additionally, redness and swelling with white moss were observed on the skin around the anus. Biopsy showed that Paget cells were diffusely present in the epithelium, and an image of squamous cell carcinoma directly under the epithelium was obtained. Taken together, the patient was diagnosed with the invasion of anal canal cancer with PS to the skin, and we performed laparoscopic abdominoperineal resection and skin carcinoma resection in the perineum. The histopathological analysis showed adenocarcinoma invading the external anal sphincter and subcutaneous adipose tissue in the vicinity of the pectinate line of the anal canal. Pagetoid spread of the adenocarcinoma was observed in the epidermis, and the open portion was slightly invaded up to the rectal mucosa. The anal skin region of the adenocarcinoma partially continued to the hair follicles, and it was complicated by squamous cell carcinoma invading the dermis. There are a few reports of anal canal cancer with PS, and the coexistence of adenocarcinoma and squamous cell carcinoma, as seen in the present case, is rare. We report our case together with relevant literature.

## 1. Introduction

Extramammary Paget's disease (EMPD) is classified into primary and secondary forms. Primary extramammary Paget's disease is a skin disease in which carcinoma cells from skin appendages such as the perineum, perianal area, scrotum, penis, and axilla invade the epidermis. Extramammary Paget's disease presents with erythematous plaques in elderly men and women [1]. Secondary EMPD with pagetoid spread (PS) is tumor invasion into neighboring organs around the epidermis due to intraepithelial progression [2]. Extramammary Paget's disease is a rare condition, and a population-based study reported an incidence rate of 0.11 per 100,000 person-years [1]. There are only a few reports on the coexistence of EMPD with squamous cell carcinoma [3, 4], and the coexistence of squamous cell carcinoma with secondary EMPD has not been reported. Herein, we report a rare case of anal canal cancer with PS, accompanied by squamous cell carcinoma.

## 2. Case Presentation

A 91-year-old man presented to our hospital with a chief complaint of anal pain for 3 years. In another hospital, *Candida* infection was discovered in the anal skin of the patients, and ketoconazole was topically applied once a day. The patient was diagnosed with a node and erythema in the anal area. His medical history included anxiety disorder and chronic gastritis, and his medication history revealed that he had been on a gastric mucosa protectant for at least one year. His family medical history was not significant.

Four years ago, the patient became aware of rashes in the anal area and received treatment at a nearby clinic. As there was no improvement, the patient was referred to the Department of Dermatology at our hospital. A biopsy of the node around the anus showed atypical cells developing as Paget's disease in the epidermis. Immunohistochemical analysis showed cytokeratin 7 (CK7) positivity, cytokeratin 20 (CK20) positivity, and gross cystic disease fluid protein 15 (GCDFP15) negativity. Tumor invasion with PS from the anus to the skin was suspected, and the patient was referred to our department for a close examination and surgical treatment.

**Figure 1:**
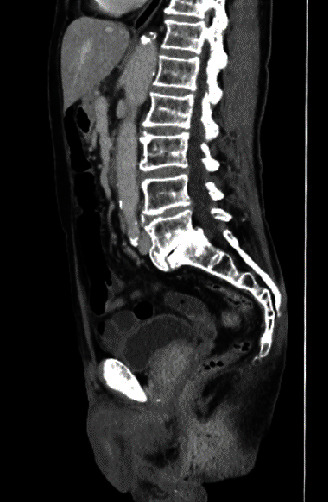
Contrast-enhanced abdominal CT scan. Sagittal section. Enhanced abdominal CT shows a contrast effect on the perianal skin in the early phase.

**Figure 2:**
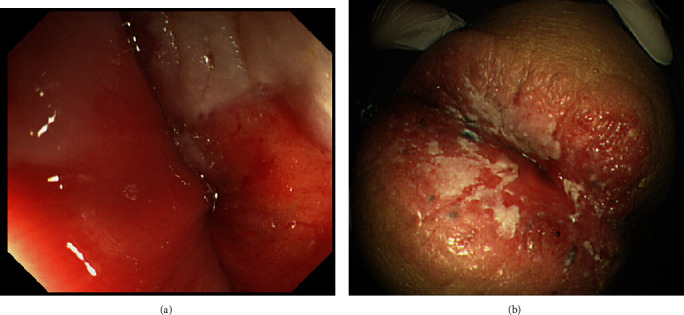
Endoscopic photograph of the lower gastrointestinal tract. (a) The mucous membrane of the anal canal was edematous and bled easily. (b) Redness and swelling with white moss were observed in the anus.

The patient was 159 cm tall and weighed 47.8 kg, with a body mass index of 18.9 kg/m^2^. Erythema (8 cm × 6 cm) was accompanied by erosion around the anus. No mass was detected in rectal examination. There were no swollen lymph nodes on either side of the groin. Laboratory blood data on admission were not significant. Regarding contrast-enhanced abdominal CT findings, in the early phase, contrast enhancement was observed in the skin around the anus, but there was no contributory finding in the intestinal tract between the anal canal and anal opening (Figure 1). Neither swelling of the inguinal lymph node nor distant metastasis was observed in the peritoneal cavity. Pelvic MRI did not reveal a clear mass or swollen lymph node in the anal area. Upper gastrointestinal endoscopy was noncontributory. Lower gastrointestinal endoscopy revealed no protruding lesion in the area between the anal canal and rectum. The mucosa of the anal canal was edematous and hemorrhagic (Figure 2(a)). Redness and swelling with white moss were observed in the anus (Figure 2(b)). Biopsy showed mammary/tubular adenocarcinoma and squamous cell carcinoma with atypical epithelium, with a tendency for keratinization in a sheet-like manner in the anal canal. In the anus, an atypical stratified squamous epithelium continuing to the outer mucosal epithelial layer grew to the funiculi in a sheet-like manner. Pagetoid cells were diffusely present in the epithelium, and an image of squamous cell carcinoma directly under the epithelium was obtained.

To cure the invasion of the skin by anal canal cancer with PS and squamous cell carcinoma of the skin, we performed laparoscopic abdominoperineal resection, lymphadenectomy, and skin carcinoma resection in the perineum. Neither lateral lymph node dissection nor inguinal lymph node dissection was performed. The skin around the anus was resected approximately 1.5 cm outside the erythema, the skin subcutaneous adipose tissue of the anus was removed, and the wound was closed without a skin flap.

**Figure 3:**
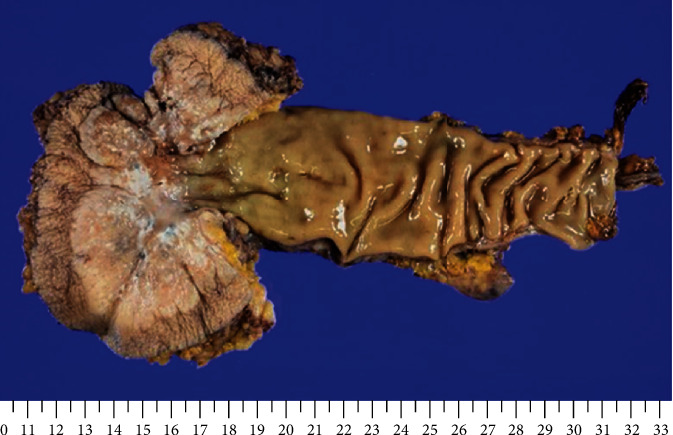
Macroscopic image of the resected specimen surrounding the anus. There was a 92 mm × 48 mm tumor accompanied by slightly protruding white moss in the entire circumference of the anus with a clear boundary between the tumor and the surrounding healthy epithelium.

For pathological examination, the resected specimens were used after formalin fixation. There was a 92 mm × 48 mm tumor accompanied by slightly protruding white moss in the entire circumference of the anus with a clear boundary between the tumor and the surrounding healthy epidermis (Figure 3).

**Figure 4:**
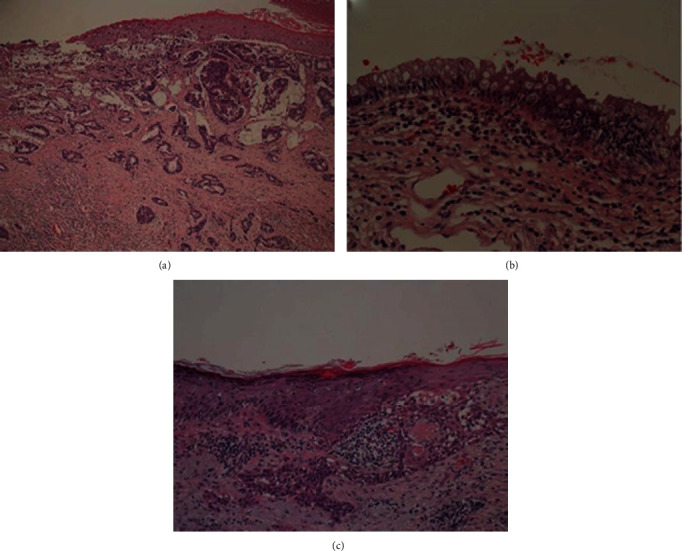
Histological images. (a) Histological image of the vicinity of the pectinate line of the anal canal showing the invasion of adenocarcinoma with tube formation directly under the stratified squamous epithelium. It was mucinous. Pagetoid cells, which are large clear cells, diffused into the stratified squamous epithelium. (b) Histological image of the area between the pectinate line and the opening on the rectal mucous side. Pagetoid spread of atypical cells was observed in the rectal epithelium. (c) A histological image of the squamous cell carcinoma around the anus. The anal skin region partially continued to the hair follicles, and it was complicated with well-differentiated squamous cell carcinoma invading the dermis.

The histological findings revealed that an adenocarcinoma invaded the external anal sphincter and subcutaneous adipose tissue in the vicinity of the pectinate line of the anal canal (Figure 4(a)). Pagetoid spread of adenocarcinoma from the anal gland was observed in the epidermis. Pagetoid cells, which are large clear cells, grew in simplex and alveolar patterns in the epidermis. The open portion of the anal canal was slightly invaded up to the rectal mucosa (Figure 4(b)). The anal adenocarcinoma skin region partially continued to the hair follicles and was complicated by squamous cell carcinoma invading the dermis (Figure 4(c)).

**Figure 5:**
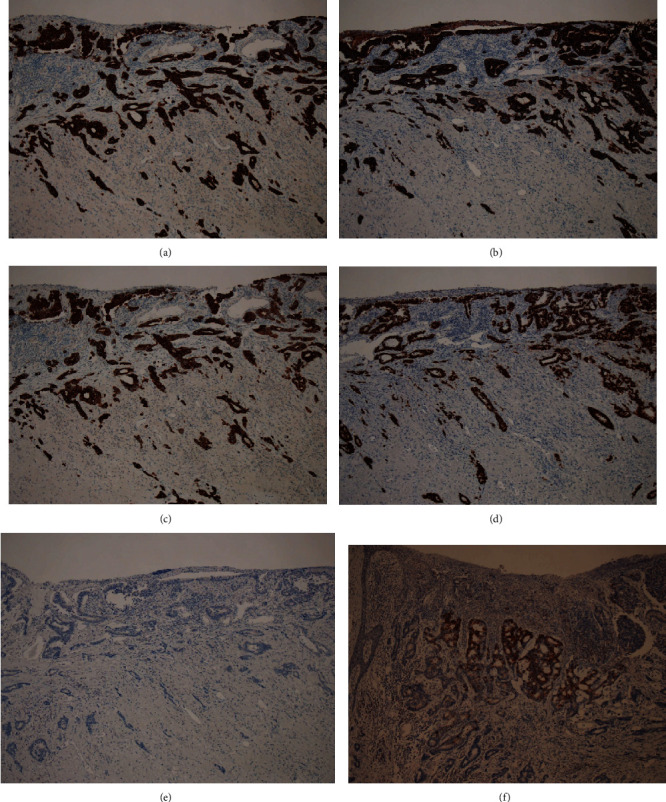
Immunohistochemical analysis of the resected adenocarcinoma around the anus. (a) Immunostaining for CK7. The adenocarcinoma directly under the squamous epithelium and atypical cells developing in the squamous epithelium was positive for CK7. (b) Immunostaining for CK19. The adenocarcinoma directly under the squamous epithelium and atypical cells developing in the squamous epithelium was positive for CK19. (c) Immunostaining for CK20. The adenocarcinoma directly under the squamous epithelium and atypical cells developing in the squamous epithelium was positive for CK20. (d) Immunostaining for CDX2. The adenocarcinoma directly under the squamous epithelium and atypical cells developing in the squamous epithelium was positive for CDX2. (e) Immunostaining of GCDFP15. The adenocarcinoma directly under the squamous epithelium and atypical cells developing in the squamous epithelium was negative for GCDFP15. (f) Immunostaining for MUC5A. The adenocarcinoma directly under the squamous epithelium was partially positive for MUC5A.

**Figure 6:**
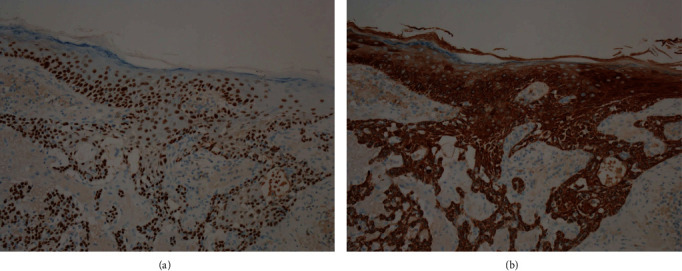
Immunohistochemical analysis of the squamous cell carcinoma in the anal skin region. (a) Immunostaining for p63. The atypical squamous epithelium was positive for p63 in all layers. Pagetoid cells diffusely present in the epithelium were negative. (b) Immunostaining for CK5/6. The atypical squamous epithelium was positive for CK5/6 in all layers. Pagetoid cells diffusely present in the epithelium were negative.

Immunohistochemical analysis of the resected tumor around the anus revealed that the adenocarcinoma was positive for CK7, Cytokeratin 19 (CK19), CK20, and CDX2 (Figures 5(a)–5(d)), partially positive for MUC5A (Figure 5(f)), and negative for GCDFP15 (Figure 5(e)). Squamous cell carcinoma was positive for p63 (Figure 6(a)) and CK5/6 (Figure 6(b)) in all layers from the fundus to the surface layer.

Although the patient developed ileus after surgery, he improved and was discharged on postoperative day 37. The patient did not require postoperative chemotherapy. He was an outpatient at our department and was being followed up. One year later, multiple liver metastases, lung metastases, and inguinal lymph node metastases were observed. He received radiation therapy (20 Gy/5 fractions) to relieve inguinal pain and pain associated with lower leg edema due to inguinal lymph node metastases. Unfortunately, the patient died 1 year and 6 months after surgery without local recurrence in the perianal area.

## 3. Discussion

Paget's disease was reported in 1874 by Sir James Paget as lesions on the nipple preceding breast cancer [5]. Currently, Paget's disease is classified into mammary Paget's disease, which occurs in the breast, and EMPD, which occurs in regions other than the breast. In addition, EMPD is classified into the primary and secondary forms. Although the clinical histopathological images of both diseases are very similar, they significantly differ in therapy and prognosis; thus, differentiation between the disease forms is important [6, 7]. Both disease forms have Paget cells around the anus in common, whereas the primary lesions are pathologically different. In primary EMPD of the skin, invasion is usually only intraepidermal, and a relatively favorable prognosis can be expected; whereas, in secondary EMPD, due to the presence of invasive carcinoma of the neighboring organs, the prognosis is usually poor [8]. Tran et al. reported that one of the treatment options is neoadjuvant radiation therapy with chemotherapy before surgery and then pelvic exenteration, which achieves a complete response [9]. Katerji et al. reported an HPV-related anorectal adenocarcinoma arising in a tubulovillous adenoma [10]. However, in our case, the immunochemical analysis result was negative for HPV.

Immunostaining has been reported to be useful in differentiating these two disease forms. In normal tissues, immunohistochemical staining for GCDFP15 is positive in the sweat gland, mammary gland, and salivary gland and negative in the gastrointestinal tract [11]. Immunohistochemical staining for CK20 is positive in the gastrointestinal epithelium, urinary bladder epithelium, and Merkel cells in normal tissues [12]. A study reported that primary EMPD of the skin showed GCDFP15 positivity and CK20 negativity, whereas PS showed GCDFP15 negativity and CK20 positivity [13]. In our patient, the preoperative skin biopsy specimens and resected tissues around the anus showed GCDFP15 negativity (Figure 5(e)) and CK20 positivity (Figure 5(c)). Thus, we considered secondary EMPD with PS. In addition, the results showed CDX2 positivity (Figure 5(d)). A study reported that in EMPD, the CDX2 positivity rate is 2%, whereas that in a Paget phenomenon of rectal anal canal cancer is 80%; thus, CDX2 may be useful for differentiating the two disease forms [14]. The present case was that of primary adenocarcinoma of the anus. Primary adenocarcinoma of the anus is classified into three types, namely, adenocarcinoma derived from the rectal mucosa, anal gland, and anal fistulas. Rectal-type carcinoma occurring in the mucosa of the anal canal and rectum is the most common, and in carcinoma originating from the anal gland, the lesion is primarily located in the wall of the anal canal, with hardly any cancer tissue in the mucosa. Immunohistochemistry is also useful for differentiating these carcinomas. Rectal-type carcinoma showed CK7 negativity, CK19 positivity, and MUC5AC negativity, whereas the carcinoma derived from the anal gland showed CK7 positivity, CK19 positivity, and MUC5AC positivity [15, 16]. The present case was positive for CK7 (Figure 5(a)), CK19 (Figure 5(b)), and MUC5AC (Figure 5(f)), which was similar to the pattern of anal gland type carcinoma. Furthermore, atypical squamous epithelial cells with para-keratocysts and pseudokeratocysts continuing to the hair follicles were observed, and the present case was complicated with squamous cell carcinoma. Squamous cell carcinoma was p63-positive and CK5/6-positive in all layers (Figures 6(a) and 6(b)), and there was a slight invasion of the dermis. p63 and CK5/6 are well-known markers of squamous cell carcinoma [17]. Based on the results of immunohistochemistry, our patient was diagnosed with coexisting multiple adenocarcinoma and squamous cell carcinoma in the perianal region, and not squamous metaplasia of adenocarcinoma. It is important to clarify the differential diagnosis between primary and secondary Paget's diseases and identify the cause of secondary Paget's disease using locally resected specimens by immunohistochemical analysis to determine the appropriate therapy.

Adjuvant radiation therapy has also been recommended, especially in multifocal diseases, lymph node metastasis, positive surgical margins, and associated malignancies [9]. Hata et al. [18] published the outcomes of eight patients with EMPD with lymph node metastasis treated with adjuvant radiation therapy. The total dose of 45–61.2 Gy (median, 59.4 Gy) was administered in 25–34 fractions (median, 33 fractions). These fractions represented a total of 43 metastatic lymph nodes. Only one node showed progression at the median follow-up time of 22 months, and the 2-year local control rate was 86% in all patients and 98% in all metastatic lymph nodes. In our case, the dose was low because the patient wanted palliative treatment.

In our literature search, we identified four cases of Paget's disease/EMPD complicated by invasive squamous cell carcinoma [3, 19, 20]. Two of these cases were squamous metaplasia occurring in Paget's disease/EMPD [19, 20], and one was secondary EMPD where squamous cell carcinoma showed pagetoid spread [4]. There is only one case report of the coexistence of adenocarcinoma and squamous cell carcinoma in EMPD [3]. There are three possible reasons why Paget's disease may be associated with squamous cell carcinoma: (1) the two tumors may occur independently, (2) SCC may be a secondary Paget's disease with pagetoid spread, and (3) phenotypic changes may have occurred during the progression of Paget's disease. In the present case, two completely different morphological or immunohistological cancers might have occurred independently at the same site, making this a case of double cancer. However, there have been no reports of cases in which PS or secondary Paget's disease is complicated by invasive squamous cell carcinoma. Although the clinical significance of squamous cell carcinoma of the skin complicating PS is unclear, further cases are expected to accumulate.

## 4. Conclusions

In the present case, we performed immunostaining before surgery and diagnosed the patient with anal canal cancer with PS complicated with squamous cell carcinoma. Laparoscopic abdominoperineal resection and malignant skin tumor resection were performed for complete cure. In addition to primary adenocarcinoma of the anal gland, squamous cell carcinoma was conceivably derived from the outer hair follicles. As there have been no case reports of anal canal cancer with PS complicated by squamous cell carcinoma, the present case is, thus, extremely rare.

## Data Availability

All data generated or analyzed during this study are included in this published article.
